# Assessing the Hemodynamic Impact of Anterior Leaflet Laceration in Transcatheter Mitral Valve Replacement: An *in silico* Study

**DOI:** 10.3389/fcvm.2022.869259

**Published:** 2022-06-09

**Authors:** Keshav Kohli, Zhenglun Alan Wei, Vahid Sadri, Andrew W. Siefert, Philipp Blanke, Emily Perdoncin, Adam B. Greenbaum, Jaffar M. Khan, Robert J. Lederman, Vasilis C. Babaliaros, Ajit P. Yoganathan, John N. Oshinski

**Affiliations:** ^1^Wallace H. Coulter Department of Biomedical Engineering at the Georgia Institute of Technology and Emory University, Atlanta, GA, United States; ^2^Department of Biomedical Engineering, University of Massachusetts Lowell, Lowell, MA, United States; ^3^Department of Radiology, St. Paul’s Hospital, The University of British Columbia, Vancouver, BC, Canada; ^4^Structural Heart and Valve Center, Emory University Hospital, Atlanta, GA, United States; ^5^Cardiovascular Branch, Division of Intramural Research, National Heart Lung and Blood Institute, National Institutes of Health, Bethesda, MD, United States; ^6^Department of Radiology and Imaging Science, Emory University School of Medicine, Atlanta, GA, United States

**Keywords:** transcatheter mitral valve replacement (TMVR), LAMPOON, left ventricular outflow tract obstruction, neo-LVOT, computational fluid dynamics (CFD), computed tomography (CT), personalized computational modeling

## Abstract

**Background:**

A clinical study comparing the hemodynamic outcomes of transcatheter mitral valve replacement (TMVR) with vs. without Laceration of the Anterior Mitral leaflet to Prevent Outflow Obstruction (LAMPOON) has never been designed nor conducted.

**Aims:**

To quantify the hemodynamic impact of LAMPOON in TMVR using patient-specific computational (*in silico*) models.

**Materials:**

Eight subjects from the LAMPOON investigational device exemption trial were included who had acceptable computed tomography (CT) data for analysis. All subjects were anticipated to be at prohibitive risk of left ventricular outflow tract (LVOT) obstruction from TMVR, and underwent successful LAMPOON immediately followed by TMVR. Using post-procedure CT scans, two 3D anatomical models were created for each subject: (1) TMVR with LAMPOON (performed procedure), and (2) TMVR without LAMPOON (virtual control). A validated computational fluid dynamics (CFD) paradigm was then used to simulate the hemodynamic outcomes for each condition.

**Results:**

LAMPOON exposed on average 2 ± 0.6 transcatheter valve cells (70 ± 20 mm^2^ total increase in outflow area) which provided an additional pathway for flow into the LVOT. As compared to TMVR without LAMPOON, TMVR with LAMPOON resulted in lower peak LVOT velocity, lower peak LVOT gradient, and higher peak LVOT effective orifice area by 0.4 ± 0.3 m/s (14 ± 7% improvement, *p* = 0.006), 7.6 ± 10.9 mmHg (31 ± 17% improvement, *p* = 0.01), and 0.2 ± 0.1 cm^2^ (17 ± 9% improvement, *p* = 0.002), respectively.

**Conclusion:**

This was the first study to permit a quantitative, patient-specific comparison of LVOT hemodynamics following TMVR with and without LAMPOON. The LAMPOON procedure achieved a critical increment in outflow area which was effective for improving LVOT hemodynamics, particularly for subjects with a small neo-left ventricular outflow tract (neo-LVOT).

## Introduction

Left ventricular outflow tract (LVOT) obstruction is a prevalent and potentially fatal complication of transcatheter mitral valve replacement (TMVR) caused by displacement of the anterior leaflet toward the ventricular septum (1). Laceration of the Anterior Mitral leaflet to Prevent Outflow Obstruction (LAMPOON) is a catheter-based technique designed to alleviate the risk of obstruction by mimicking surgical anterior leaflet resection. The optimal result of the LAMPOON procedure is a complete midline laceration of the anterior leaflet which, following TMVR, exposes open cells of the transcatheter valve. These exposed cells are thought to permit additional blood flow through the LVOT and decrease the risk of obstruction (2). LAMPOON is considered for patients who have a predicted residual LVOT neo-left ventricular outflow tract (neo-LVOT) area <200 mm^2^ and are therefore anticipated to have significant LVOT obstruction from TMVR (3).

Despite the growing clinical experience with this technique, a controlled clinical trial comparing the outcomes of patients undergoing TMVR with vs. without LAMPOON has never been designed nor conducted. It would be impossible to conduct such a trial in which the control intervention (i.e., TMVR without LAMPOON) would be anticipated to cause immediate LVOT obstruction following valve implantation. Thus, while the LAMPOON technique has been shown to be safe and feasible, its impact on left ventricular outflow hemodynamics has never been investigated.

For this purpose, we conducted a computational (*in silico*) controlled study of TMVR with LAMPOON vs. TMVR without LAMPOON. We used a validated *in silico* approach using post-procedure computed tomography (CT) scans from subjects at prohibitive risk of LVOT obstruction who successfully underwent TMVR with LAMPOON (4). 3-D anatomical and flow information were extracted from the CT datasets and used as inputs into personalized computational fluid dynamics (CFD) models. For each subject, hemodynamics of TMVR with and without LAMPOON were simulated and compared. We *hypothesized* that LAMPOON improves flow dynamics through the LVOT in patients with a small neo-LVOT.

## Materials and Methods

### LAMPOON Trial Dataset

De-identified subject data from the LAMPOON investigational device exemption trial (NCT03015194) were retrospectively evaluated under an Institutional Review Board approved protocol (4). All subjects in that trial (*N* = 30) were considered at prohibitive risk for TMVR-related LVOT obstruction based on a predicted end-systolic neo-LVOT area of <200 mm^2^. Subjects underwent a valve-in-ring or valve-in-mitral annular calcification procedure with the SAPIEN 3 valve (Edwards Lifesciences, Irvine, CA, United States) with retrograde LAMPOON prior to valve implantation. The LAMPOON technique and 30-day clinical outcomes have been previously reported (2, 4). Briefly, this technique involves three main steps: (1) anterior leaflet traversal with a guidewire, followed by (2) leaflet laceration by electrifying and pulling the guidewire from base to tip, immediately followed by (3) TMVR. Complete details on the utilized retrograde technique and newer LAMPOON technique iterations were recently described (5).

### Selection of Study Cohort

Inclusion criteria for this study were: (1) the availability of a post-procedure, multi-phase CT scan with contrast-enhancement, and (2) a splayed anterior leaflet that was visible on post-procedure CT and exposed cells of the implanted transcatheter valve. Of the thirty subjects in the LAMPOON investigational device exemption trial, sixteen subjects did not have adequate visualization of the anterior mitral leaflet on the post-procedure CT scan due to image quality, and two subjects did not have an available post-procedure CT due to patient death. Of the remaining twelve subjects, four had CTs in which we could not visualize exposed cells of the transcatheter valve, despite a successful anterior leaflet laceration. Thus, eight subjects were selected for the present study who had a splayed anterior leaflet which exposed open cells of the implanted transcatheter valve, as verified on post-procedure CT. These eight subjects were at risk of a *fixed* LVOT obstruction from TMVR due to a small predicted neo-LVOT, and not a *dynamic* obstruction due to a long anterior leaflet.

### Post-procedure Computed Tomography Datasets

Contrast-enhanced, multi-phase CT scans were acquired after the procedure, either pre-discharge or within 30-days of follow-up. CT images were reconstructed in 10% intervals over the cardiac cycle within <1.0-mm slice thickness. Mimics 20.0 (Materialize, Leuven, Belgium) image post-processing software was used to generate 3-D models of the left ventricle and aorta using previously described segmentation techniques (6). The left ventricular volume was segmented from the CT images for all phases across the cardiac cycle, and the time rate of change in volume was calculated to provide time-varying flow rate. This method for deriving flow rate from left ventricular volumes has been described previously (7).

### 3D Models Created for Each Subject

**FIGURE 1:**
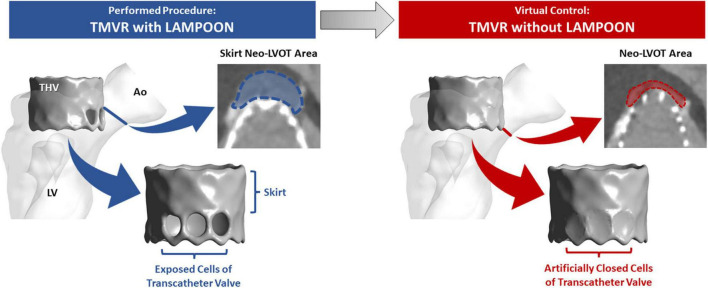
3D anatomical models created for each subject. The left model represents the actual performed procedure (TMVR with LAMPOON). In this model, the left ventricular anatomy and implanted transcatheter valve geometry were segmented directly from the post-procedure CT dataset. The number and location of transcatheter valve cells exposed by LAMPOON were modeled based on visualization of the anterior leaflet splay in the post-procedure CT dataset. To simulate a “virtual control” procedure in which LAMPOON was not performed (TMVR without LAMPOON), we *artificially* closed the exposed cells of the transcatheter valve as shown in the right model. Ao, aorta; LV, left ventricle; THV, transcatheter heart valve.

Two different 3D models were created for each subject using the post-procedure CT datasets. The first model represented “TMVR with LAMPOON.” In this model, the *actual* implanted transcatheter valve and splayed anterior leaflet were reconstructed in 3D from the post-procedure CT (Figure 1, left). These reconstructions of the implanted valve therefore incorporated the *actual* valve deployment depth, angulation, and expansion characteristics. The positions of transcatheter valve cells exposed by LAMPOON were identified visually on the post-procedure CT, and these exposed cells were intentionally left patent in this model.

The second model represented “TMVR without LAMPOON.” In this model, the exposed cells of the transcatheter valve were *artificially closed*, mimicking a completely intact anterior leaflet geometry (Figure 1, right). Since the model of “TMVR with LAMPOON” mimicked the performed interventional procedure, the model of “TMVR without LAMPOON” was considered to mimic a control “virtual procedure.” Both models were designed to be anatomically identical other than the presence or absence of the splayed anterior leaflet.

### Measurement of Neo-Left Ventricular Outflow Tract and Skirt Neo-Left Ventricular Outflow Tract Area

Computed tomography datasets were utilized to measure the neo-LVOT and skirt neo-LVOT area. The neo-LVOT area was measured in a plane perpendicular to the LVOT and located at the narrowest point along the residual LVOT after TMVR (Figure 1, right). Following LAMPOON, the transcatheter valve skirt still protrudes into the LVOT, creating a narrowing at the transcatheter valve skirt called the “skirt” neo-LVOT (8). Figure 1 illustrates differences between the neo-LVOT and skirt neo-LVOT. The skirt neo-LVOT area was measured in a plane perpendicular to the LVOT and located at the level of the transcatheter valve skirt (Figure 1, left). Both neo-LVOT and skirt neo-LVOT area measurements were first predicted on baseline CT, and then measured on post-procedure CT.

### Computational Fluid Dynamics Simulations

Computational fluid dynamics (CFD) is a method used to simulate fluid flow with computer modeling. In this study, we utilized a validated CFD workflow for simulating patient-specific LVOT hemodynamics based on cardiac CT data (7). Patient-specific CFD simulations were performed for both modeled conditions (TMVR with and without LAMPOON) for each subject. The output of these simulations provided spatially varying velocity and pressure data across the entire left ventricle and aorta. The workflow used for performing the CFD simulations has been previously described and validated against clinical measurements from Doppler echocardiography (7). The average difference between CFD-derived and echocardiography-derived peak LVOT velocity measurements was <10%, indicating a good agreement between the CFD simulations and clinical measurements. This CFD workflow is briefly summarized below.

All CFD simulations were performed for the peak-systolic phase of the cardiac cycle. The cardiac phase of the post-procedure CT scan which most closely coincided with the peak systolic flow rate was selected for creating a 3D model of the left heart anatomy and implanted transcatheter valve. For each 3D model, an inlet was placed at the left ventricular apex, and an outlet was placed at the sino-tubular junction. 3-Matic 12.0 (Materialize, Leuven, Belgium) computer aided design software was used to place a virtual wall at the level of the bioprosthetic valve leaflets to prevent regurgitant flow. A volumetric mesh was then generated using ANSYS Fluent Meshing (Ansys, Inc. Canonsburg, PA, United States). This mesh included polyhedral elements with an edge length of 0.5 mm, and a prismatic boundary layer mesh (10 layers with geometric growth of 1.05) which was applied on the left ventricular wall. The total number of mesh elements per patient model was on average ∼1,800,000 elements. Transition-to-turbulence characteristics in the flow field were accounted for using the scale-adaptive simulation turbulence model. Turbulence boundary conditions included (1) hydraulic diameter (which is the inlet diameter, *D*_*H*_) and (2) turbulence intensity (approximated using 0.16 × *Re*_*D*_^–0.125^, where *Re*_*D*_ is the Reynolds number based on the patient-specific flow rate and the hydraulic diameter).

The time-varying left ventricular volume curve (obtained from the post-procedure CT scan) was used to calculate a peak systolic flow rate, which was then prescribed as the inlet boundary condition. The outlet boundary condition was set to a zero-reference pressure. All structures were assumed to be rigid and non-permeable, and a no-slip boundary condition was applied at the walls. Blood was modeled as a single-phase Newtonian fluid with a density of 1060 kg/m^3^ and dynamic viscosity of 0.0034 poise. The 3-D Navier-Stokes equations were solved in ANSYS Fluent 19.0 using the SIMPLE scheme for pressure-velocity coupling. A bounded second-order implicit transient formulation was utilized with a time step of 0.001 s. The solution was considered converged when residuals in momentum and turbulence variables declined below 10^–4^. Three-thousand time steps were simulated, with the last time step being used for data analysis.

### Obtaining Simulated Hemodynamics

Hemodynamic metrics were extracted from each CFD simulation, including the peak LVOT velocity (*V*_*peak*_), peak LVOT pressure gradient (Δ*P*_*peak*_), and peak LVOT effective orifice area (*EOA*_*peak*_). To calculate Δ*P*_*peak*_, the ventricular pressure was derived from an average pressure across the model inlet (LV apical plane), and the aortic pressure was derived from an average pressure across the model outlet (sino-tubular junction plane). Δ*P*_*peak*_ was then calculated as the difference between the ventricular and aortic pressures. *EOA*_*peak*_ was calculated as the ratio of the peak volumetric flow rate (*Q*_*peak*_) to the peak outflow velocity (*V*_*peak*_) (9).

### Data Analysis

All data analyses were performed with the MedCalc statistical software package (MedCalc, Ostend, Belgium). Simulated hemodynamics for each subject were compared between the two modeled conditions (TMVR with and without LAMPOON) using the paired samples *t*-test or Wilcoxon test for parametric and non-parametric data, respectively. Statistical significance was defined as *p* < 0.05.

## Results

### Subject Characteristics

Baseline, procedural and post-procedural characteristics are shown in Table 1. All subjects were considered to be at prohibitive risk of LVOT obstruction based on small ventricular anatomy and small LVOT clearance (end-systolic neo-LVOT area <200 mm^2^ as predicted on baseline CT), and therefore were considered candidates for the LAMPOON procedure. All subjects included were female, likely due to the selection of patients with small ventricular anatomy for LAMPOON. The indication for mitral valve replacement was mitral regurgitation in three subjects, and mitral stenosis in five subjects.

**TABLE 1:** Baseline, procedural, and post-procedural characteristics.

	Subject 1	Subject 2	Subject 3	Subject 4	Subject 5	Subject 6	Subject 7	Subject 8
**Baseline characteristics**								
Age, years	85	82	50	75	84	80	83	61
Gender	Female	Female	Female	Female	Female	Female	Female	Female
Ejection fraction, %	61	65	56	53	54	67	67	70
LVEDV, mL from echo	75	56	48	40	36	78	65	27
Body surface area, m^2^	1.73	1.81	1.77	1.68	1.57	1.99	1.8	1.43
Mitral pathology	MR	MS	MS	MS	MS	MR	MR	MS
Aortic stenosis	None	Mild	None	None	None	None	None	Mild
Neo-LVOT area at end-systole (predicted), mm^2^	111	57	143	81	108	168	59	55
Skirt neo-LVOT area at end-systole (predicted), mm^2^	241	278	210	247	236	333	216	189
**Procedural characteristics**								
TMVR setting	Valve-in-Ring	Valve-in-MAC	Valve-in-Ring	Valve-in-MAC	Valve-in-MAC	Valve-in-MAC	Valve-in-MAC	Valve-in-MAC
Successful leaflet laceration	Yes	Yes	Yes	Yes	Yes	Yes	Yes	Yes
Successful TMVR	Yes	Yes	Yes	Yes	Yes	Yes	Yes	Yes
Implanted SAPIEN size, mm	26	29	23	29	29	29	29	29
No. of THV cells exposed by splayed leaflet	1.5	2	2	1	3	2	2.5	2
Combined area of THV cells exposed by splayed leaflet, mm^2^	41	86	55	45	87	88	88	70
Deployment depth, % in LV	53	77	46	85	71	58	59	39
Para-valvular regurgitation	None	Mild	None	Trace	Mild	None	None	Trace
**Post-procedural characteristics**								
Time of post-procedure CT scan	30-day	30-day	Pre-discharge	Pre-discharge	30-day	30-day	Pre-discharge	30-day
Post-procedure peak flow rate, L/min	13.3	18.8	21.3	15.3	20.3	37.8	24.8	30.5
Neo-LVOT area at end-systole, mm^2^	211	187	293	42	19	223	30	221
Neo-LVOT area at peak-systole, mm^2^	266	256	379	153	129	250	49	337
Skirt neo-LVOT area at end-systole, mm^2^	320	427	307	145	211	341	182	316
Skirt neo-LVOT area at peak-systole, mm^2^	344	418	351	176	231	313	162	358

*Echocardiography was used to derive ejection fraction, left ventricular end-diastolic volume, aortic stenosis severity, and para-valvular regurgitation severity. The peak flow rate was obtained from the time-varying CT-derived left ventricular volumes. CT, computed tomography; LV, left ventricle; LVEDV, left ventricular end-diastolic volume; MAC, mitral annular calcification; MR, mitral regurgitation; MS, mitral stenosis; THV, transcatheter heart valve; TMVR, transcatheter mitral valvereplacement.*

All subjects underwent successful midline anterior leaflet laceration (LAMPOON procedure) immediately followed by investigational use of the SAPIEN 3 transcatheter heart valve in the mitral position. The setting of valve replacement was valve-in-ring for two subjects, and valve-in-mitral annular calcification for six subjects. Following valve implantation, all subjects had trace or no para-valvular regurgitation. The number of transcatheter valve cells exposed by the splayed anterior mitral leaflet ranged from 1 to 3 cells. The combined area of the exposed cells ranged from 41 to 88 mm^2^, and the area of a single exposed cell was on average 36 ± 7 mm^2^. Valve deployment depth (percent deployed in left ventricle) ranged from 39 to 85%. At follow up, the peak systolic flow rate (calculated from the post-procedure CT-derived LV volumes) ranged from 13 to 38 L/min.

### Effect of LAMPOON on Simulated Left Ventricular Flow Patterns

**FIGURE 2:**
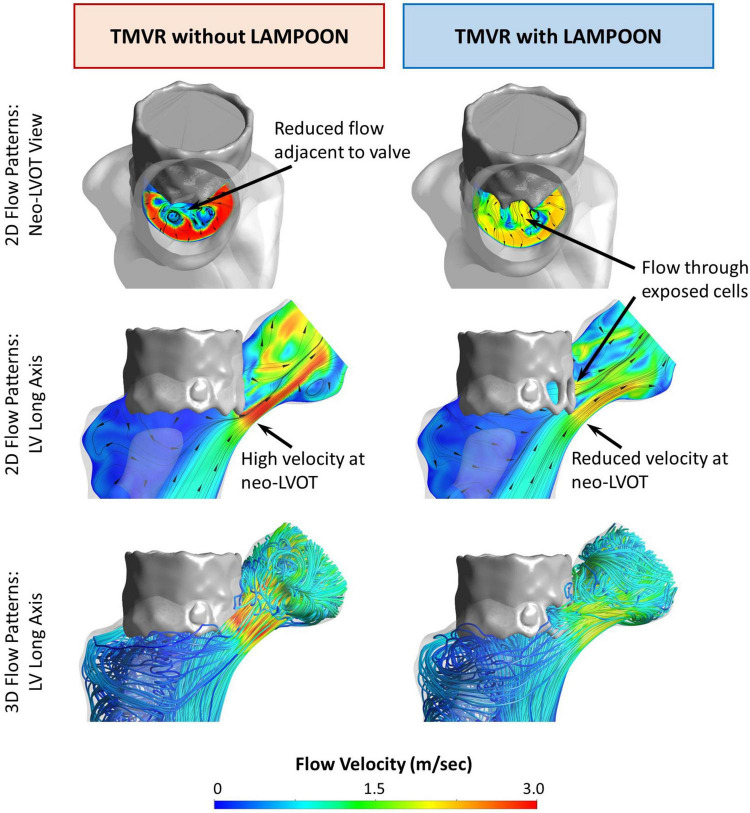
Effect of LAMPOON on left ventricular flow patterns. Simulated flow patterns for a representative subject (Subject 5) are shown in color maps (red = highest velocity, blue = lowest velocity) with streamlines. The left column shows the flow simulation for the virtual control procedure (TMVR without LAMPOON). For this condition, all flow is directed through the narrowed residual LVOT (neo-LVOT), resulting in a narrowed and high-velocity outflow jet. Additionally, there is a region of reduced flow in the immediate vicinity of the valve (upper left image). The right column shows the flow simulation for the actual performed procedure (TMVR with LAMPOON). For this condition, additional flow occurs through exposed cells of the transcatheter valve due to LAMPOON. Following LAMPOON, the velocity of flow through the neo-LVOT is reduced, and there is improved flow in the immediate vicinity of the valve (upper right image). Ao, aorta; LV, left ventricle; neo-LVOT, neo-left ventricular outflow tract; THV, transcatheter heart valve.

Simulations of left ventricular flow patterns are depicted by color maps (red = highest velocity, blue = lowest velocity) with streamlines for a representative subject (Subject 5) (Figure 2). For the condition of the virtual control procedure (TMVR without LAMPOON), flow acceleration occurred at the level of the neo-LVOT, resulting in a narrowed outflow jet (Figure 2, left panel). In this condition, the neo-LVOT was shown to be the predominant geometric narrowing for systolic flow. All forward flow was directed through the neo-LVOT, causing an increase in velocity at this location due to the reduced flow area. Additionally, there was a region of reduced flow adjacent to the transcatheter valve due to the narrowed outflow jet, as noted in the neo-LVOT short-axis view (Figure 2, upper left image).

For the condition of the performed interventional procedure (TMVR with LAMPOON), flow occurred both through the neo-LVOT and through exposed cells of the transcatheter valve (Figure 2, right panel). LAMPOON provided an additional source of flow into the LVOT, leading to a reduction in outflow velocity due to the increased flow area. The region of reduced flow adjacent to the valve in the case of TMVR without LAMPOON was decreased in the case of TMVR with LAMPOON, thus reflecting flow closer to normal flow physiology (Figure 2, upper right image). Following TMVR with LAMPOON, the main impediment to forward flow was observed to be the transcatheter valve skirt which still projected into the native LVOT.

### Effect of LAMPOON on Simulated Left Ventricular Pressure Fields

**FIGURE 3:**
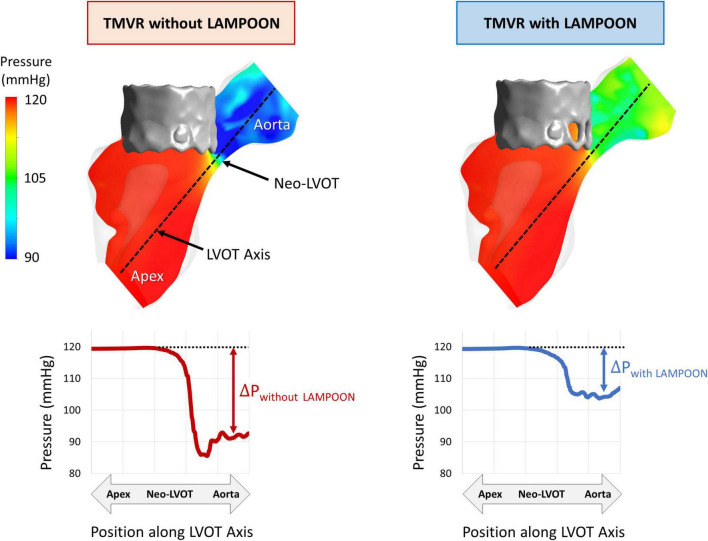
Effect of LAMPOON on left ventricular pressure fields. Simulated pressure fields for a representative subject (Subject 5) are shown in color maps (red = highest pressure, blue = lowest pressure). The left column shows the simulated pressure drop at the LVOT for the virtual control procedure (TMVR without LAMPOON). The right column shows the simulated pressure drop at the LVOT for the actual performed procedure (TMVR with LAMPOON). The simulated LVOT gradient is substantially reduced following LAMPOON. In this subject, LAMPOON exposed three cells of the transcatheter valve, corresponding to a total increase in flow area of 87 mm^2^.

Simulations of left ventricular pressure fields are depicted by color maps (red = highest pressure, blue = lowest pressure) for a representative subject (Subject 5) (Figure 3). For the condition of the virtual control procedure (TMVR without LAMPOON), there was a substantial pressure drop which occurred at the location of the neo-LVOT, corresponding to the location of geometric narrowing. For the condition of the performed interventional procedure (TMVR with LAMPOON), the simulated pressure drop was reduced at the neo-LVOT due to the increase in flow area from the splayed anterior leaflet. In this subject, the splayed anterior leaflet from LAMPOON exposed three cells of the transcatheter valve, corresponding to a total increase in flow area of 87 mm^2^.

### Comparison of Simulated Hemodynamics

**FIGURE 4:**
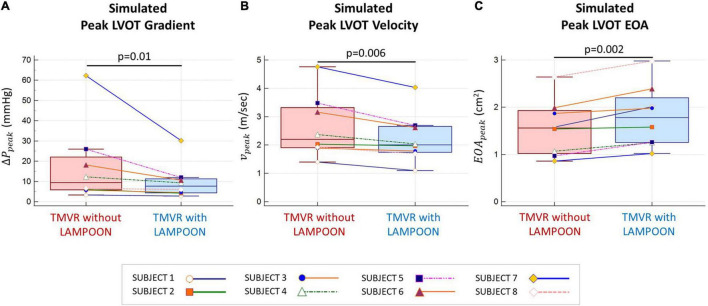
Comparison of simulated hemodynamics following TMVR with and without LAMPOON. Simulated hemodynamics following TMVR with LAMPOON were superior to those following TMVR without LAMPOON for all subjects. Following LAMPOON, the simulated peak outflow gradient (Δ*P*_*peak*_) and velocity (*v*_*peak*_) decreased in all subjects (*p* < 0.05 for both comparisons) **(A,B)**. Additionally, the simulated peak effective orifice area (*EOA*_*peak*_) increased following LAMPOON in all subjects (*p* < 0.05) **(C)**. LVOT, left ventricular outflow tract.

As compared to TMVR without LAMPOON, TMVR with LAMPOON resulted in lower *V*_*peak*_, lower Δ*P*_*peak*_, and higher *EOA*_*peak*_ by 14 ±7% (*p* = 0.006), 31 ±17% (*p* = 0.01), and 17 ±9% (*p* = 0.002), respectively (Figure 4). Following LAMPOON, *V*_*peak*_ decreased from 2.6 ±1.1 m/s to 2.2 ±0.9 m/s, Δ*P*_*peak*_ decreased from 17.5 ±19.6 mmHg to 10.0 ± 8.8 mmHg, and *EOA*_*peak*_ increased from 1.6 ±0.6 cm^2^ to 1.8 ±0.7 cm^2^ (*p* < 0.05 for all comparisons). Subject 7 had a substantially larger simulated LVOT gradient than the other subjects and could be considered an outlier (Figure 4A). However, when we excluded this subject from the analysis, we observed the same significant trend for the effect of LAMPOON on simulated LVOT gradient (*p* = 0.02).

### Relationship Between Neo-Left Ventricular Outflow Tract Area and LAMPOON Efficacy

**FIGURE 5:**
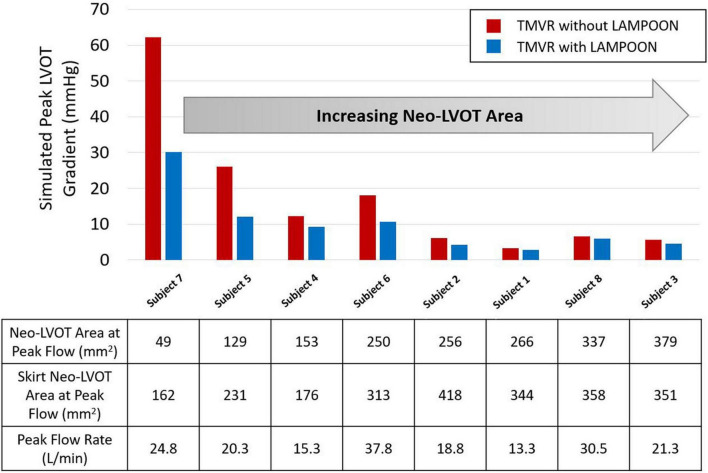
Relationship between post-procedure neo-LVOT area and hemodynamic improvement with LAMPOON. Subjects with a smaller post-procedure neo-LVOT area experienced a greater hemodynamic improvement with LAMPOON. The largest improvement in simulated LVOT gradient (32 mmHg reduction in gradient) was observed in the subject with the smallest post-procedure neo-LVOT area (49 mm^2^). As neo-LVOT area increased beyond 250 mm^2^, there was a smaller hemodynamic improvement with LAMPOON (<2 mmHg reduction in gradient). Neo-LVOT area = cross-sectional area at the narrowest point of the neo-left ventricular outflow tract. Skirt neo-LVOT area = cross-sectional area of the neo-left ventricular outflow tract measured at the level of the transcatheter valve skirt.

LAMPOON was most effective in subjects who had a small post-procedure neo-LVOT area, as measured on post-procedure CT (Figure 5). The largest hemodynamic improvement with LAMPOON was observed in Subject 7 who had the smallest post-procedure neo-LVOT area across all subjects (49 mm^2^). This subject experienced a 32 mmHg reduction in the simulated LVOT gradient following LAMPOON. 3D flow patterns in this subject were also shown to substantially improve following LAMPOON, as evidenced by more centrally directed flow along the LVOT (Supplementary Video 1).

**FIGURE 6:**
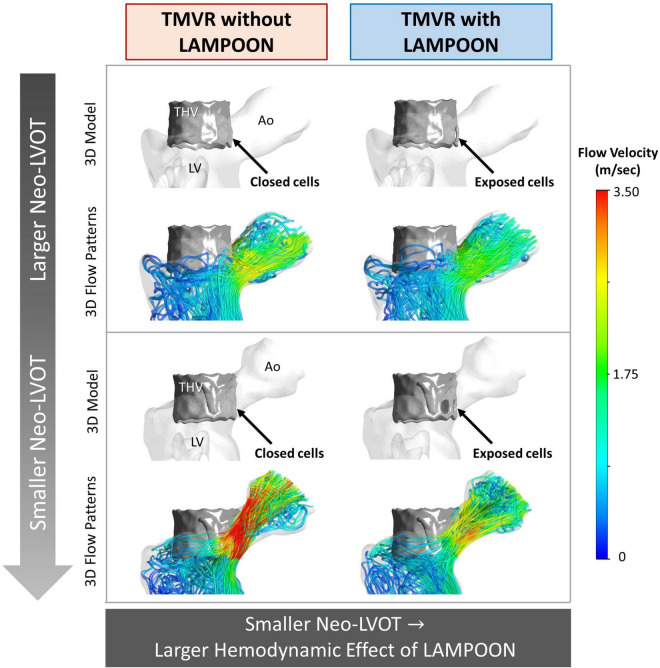
Flow simulations demonstrating the relationship between neo-LVOT size and LAMPOON efficacy. Two representative subjects are shown, where the subject shown in the top panel (Subject 6) has a larger neo-LVOT area as compared to the subject shown in the bottom panel (Subject 7). 3D models and simulated flow patterns are shown for the conditions of TMVR with and without LAMPOON. As neo-LVOT area decreases, the hemodynamic effect of LAMPOON increases. The total area of the transcatheter valve cells exposed by the splayed anterior leaflet were identical in both subjects (88 mm^2^). Ao, aorta; LV, left ventricle; neo-LVOT, neo-left ventricular outflow tract; THV, transcatheter heart valve.

As the post-procedure neo-LVOT area increased beyond 250 mm^2^, LAMPOON began to demonstrate a smaller hemodynamic effect. For all subjects with a post-procedure neo-LVOT area greater than 250 mm^2^, the simulated LVOT gradient reduced by less than 2 mmHg following LAMPOON. This finding is also illustrated in Figure 6 which shows simulations of flow patterns in two representative subjects with different neo-LVOT dimensions.

## Discussion

This study evaluated the hemodynamic impact of LAMPOON in TMVR using patient-specific *in silico* models. The main finding was that LAMPOON improved outflow hemodynamics in all subjects, with a larger hemodynamic effect in the setting of a smaller post-procedure neo-LVOT area. Because of the impossibility of performing a controlled clinical trial of TMVR with vs. without LAMPOON, the present *in silico* study is the first to provide quantitative insight into the hemodynamic effect of LAMPOON.

Left ventricular outflow tract obstruction is a significant problem for the broader adoption of TMVR as a therapy for patients with mitral valve disease who are not otherwise surgical candidates. The risk of LVOT obstruction is a primary reason for screen failure in TMVR device trials (10). For patients at prohibitive risk of obstruction, LAMPOON is a potential option to mitigate the risk of obstruction. To date, all clinical studies of LAMPOON to have been single-arm studies to assess clinical safety and feasibility of the technique (4, 11, 12).

The hemodynamic impact of LAMPOON in TMVR has not previously been investigated since patients who are considered for LAMPOON are anticipated to be at prohibitive risk of LVOT obstruction and therefore are not considered anatomical candidates for the control procedure (TMVR without LAMPOON) which would be needed to compare hemodynamic outcomes. The only way to quantitatively assess the hemodynamic impact of the LAMPOON procedure would be to perform a controlled *in vivo* (animal), *in vitro* (bench), or *in silico* (computational) study. *In vivo* studies are not always feasible due to procedural and anatomical limitations of the animal model. *In vitro* studies are more feasible but are also often very tedious to perform and can be costly. We chose to perform a controlled *in silico* study, which allowed us to use each subject as their own virtual comparator.

*In silico* modeling revealed that LAMPOON leads to a consistent improvement in hemodynamics following TMVR. Mechanistically, splaying the anterior mitral leaflet allows additional flow into the LVOT which decreases LVOT velocity and pressure gradient, while increasing the effective orifice area. The average number of transcatheter valve cells exposed by LAMPOON was 2 ±0.6 cells which corresponded to a total increase in outflow area of 70 ±20 mm^2^. On average, the area of a single exposed cell was 36 ±7 mm^2^. This small increment in outflow area led to a critical improvement in simulated hemodynamics, particularly for subjects with a smaller post-procedure neo-LVOT area (Figure 5). In this study, TMVR without LAMPOON was modeled by artificially closing the exposed transcatheter valve cells to mimic an intact anterior leaflet. However, in the clinical setting, the anterior leaflet may not only drape the cells but also hang lower beyond the valve frame. Thus, the actual improvement in hemodynamics with LAMPOON may be even more dramatic than what was calculated in this study.

Flow simulations in this study also validated the importance of the skirt neo-LVOT concept when predicting the risk of LVOT obstruction following TMVR with LAMPOON. The skirt neo-LVOT area is measured at the level of the transcatheter valve skirt, and represents the main impediment to forward flow following TMVR with anterior leaflet resection or LAMPOON (8). In the subject with the smallest post-procedure neo-LVOT area (Subject 7), there was the largest hemodynamic improvement with LAMPOON (>30 mmHg decrease in simulated LVOT gradient). However, despite this improvement, this subject still had a 30 mmHg simulated LVOT gradient post-LAMPOON due to a small skirt neo-LVOT area (162 mm^2^). While initial clinical data suggested a skirt neo-LVOT area of <150 mm^2^ should be avoided to prevent significant LVOT obstruction (catheterization gradient >30 mmHg), a more recent study from our group has shown that a skirt neo-LVOT area >180 mm^2^ is ideal to ensure survival with measurable clinical benefit (e.g., improvement in Kansas City Cardiomyopathy Questionnaire Score) (9). Thus, flow simulations in this study corroborate the clinical observation that LVOT obstruction may still occur following LAMPOON in the case of a small skirt neo-LVOT, and confirm that the skirt neo-LVOT should be routinely and carefully evaluated prior to performing TMVR with LAMPOON.

This study also highlighted that there can be differences between the *anticipated* risk of LVOT obstruction and the *actual* risk of LVOT obstruction in patients undergoing TMVR. All subjects in this study were initially thought to be at prohibitive risk for obstruction based on a predicted end-systolic neo-LVOT area of <200 mm^2^ measured on baseline CT. However, the neo-LVOT area measured on post-procedure CT was found to be substantially larger (>100 mm^2^) than the predicted neo-LVOT area at baseline in four subjects (Subjects 1, 2, 3, and 8) (Table 1), indicating that the actual risk of obstruction was substantially lower than the anticipated risk of obstruction. These four subjects experienced the least simulated hemodynamic benefit with LAMPOON. These data suggests that, in patients undergoing a valve-in-ring or valve-in-mitral annular calcification procedure, the anticipated risk of obstruction may in fact differ from the actual risk based on factors such as valve deployment depth (more atrial vs. more ventricular) or the valve deployment orientation (coaxial vs. canted with respect to the mitral valve). Personalized computational modeling may eventually help in better predicting the hemodynamic outcome of patients (13), and should account for a range of valve deployment scenarios. Recent interventional techniques have also enabled more predictable deployment of the transcatheter valve in the predicted landing zone, and may help improve future predictions of LVOT obstruction risk (14, 15).

*In silico* studies have the potential to provide key insights into the performance of novel interventional procedures such as LAMPOON which may be impossible to evaluate in a controlled clinical trial. The advantages of *in silico* studies are to (1) avoid the costs associated with conventional clinical trials, (2) avoid risk to patients, and (3) study unique metrics which may be difficult or impossible to attain in a clinical setting. Within the realm of structural heart disease interventions, prior *in silico* studies have evaluated para-valvular regurgitation in transcatheter aortic valve replacement (16, 17), device size selection for left atrial appendage occlusion (18), and LVOT hemodynamics in the setting of TMVR (7, 19, 20). In the future, *in silico* trials may also be used to supplement conventional clinical trial evidence and support the regulatory process of new devices and procedures (21). With appropriate validation, *in silico* data can represent a potentially valuable form of evidence which can be obtained at a reduced cost and without risk to patients (22).

### Limitations

This was a retrospective study with a small sample size. As adoption of the LAMPOON technique expands, larger datasets will be needed to corroborate the findings of this study. The modeling of blood flow in this study using CFD may be limited given the assumptions of fixed left ventricular walls with zero wall velocity during peak systole, and steady flow boundary conditions. Since the pressure gradient across a normal aortic valve should be negligible relative to the gradient across an obstructed LVOT, we did not model an aortic valve in our simulations for simplicity. The anterior mitral leaflet was modeled depending on whether the leaflet could be reliably segmented on the post-procedure CT dataset. The posterior mitral leaflet was not modeled since this leaflet is not affected by the LAMPOON procedure and should not impact flow in the LVOT. The value of our CFD workflow is to provide a simplified, rapid approach to simulate LVOT hemodynamics. This method has been previously validated and has demonstrated good accuracy (<10% error) when compared to clinical hemodynamic measurements (7). Despite the discussed limitations, this study provides unique insight into the hemodynamic effect of LAMPOON which would have been impossible to study in a clinical setting.

## Conclusion

A controlled *in silico* study was performed to investigate the hemodynamic impact of LAMPOON in TMVR. LAMPOON achieved a critical increment in outflow area which resulted in a consistent improvement in hemodynamic outcomes and 3D flow patterns following TMVR. Patients with a smaller post-procedure neo-LVOT area experienced a greater hemodynamic improvement with LAMPOON. This study demonstrates the potential for *in silico* studies to evaluate procedures virtually which may improve cost-effectiveness and patient safety as compared to a conventional clinical trial.

## Data Availability Statement

Data can be made available upon reasonable request to NHLBI.

## Ethics Statement

All subject data were acquired and analyzed under Institutional Review Board approvals at the National Institutes of Health (NCT03015194). The patients/participants provided their written informed consent to participate in this study.

## Author Contributions

KK, ZW, VS, RL, VB, AY, and JO: conception and design of the work. EP, AG, JK, RL, and VB: acquisition and compilation of clinical data. KK and ZW: development of methods for computer simulations. KK, ZW, AS, PB, and EP: initial drafting of manuscript. All authors analysis and interpretation of data, critical review of manuscript for important intellectual content, and final approval of manuscript.

## Conflict of Interest

KK has served as a consultant for Abbott Vascular. PB has served as a consultant for Edwards Lifesciences, Tendyne, Neovasc, and Circle Imaging. AG has served as a proctor for Edwards Lifesciences, Medtronic, and Abbott Vascular; and has served as a consultant for and is an equity holder in Transmural Systems. VB has served as a consultant for Edwards Lifesciences and Abbott Vascular; and has served as a consultant for and is an equity holder in Transmural Systems. JK and RL were co-inventors on patents, assigned to the NIH, on devices for leaflet laceration. The remaining authors declare that the research was conducted in the absence of any commercial or financial relationships that could be construed as a potential conflict of interest.

## Publisher’s Note

All claims expressed in this article are solely those of the authors and do not necessarily represent those of their affiliated organizations, or those of the publisher, the editors and the reviewers. Any product that may be evaluated in this article, or claim that may be made by its manufacturer, is not guaranteed or endorsed by the publisher.

## Abbreviations

CFD: computational fluid dynamics
CT: computed tomography
LAMPOON: intentional laceration of the anterior mitral leaflet to prevent left ventricular outflow tract obstruction
LVOT: left ventricular outflow tract
TMVR: transcatheter mitral valve replacement.
